# Images in cardiovascular disease. The right type of single coronary artery

**DOI:** 10.1186/s44348-024-00007-4

**Published:** 2024-06-12

**Authors:** Tae Il Yang, Minsu Kim, Jung Han Hwang, Mi-Seung Shin

**Affiliations:** 1 Division of Cardiology, Department of Internal Medicine, Gil Medical Center, Gachon University College of Medicine, 21 Namdong-Daero 774Beon-Gil, Namdong-Gu, Incheon, 21565 Korea; 2 Department of Radiology, Gil Medical Center, Gachon University College of Medicine, Incheon, Korea

**Keywords:** Coronary artery, Computed tomography angiography, Dyspnea

**Fig. 1:**
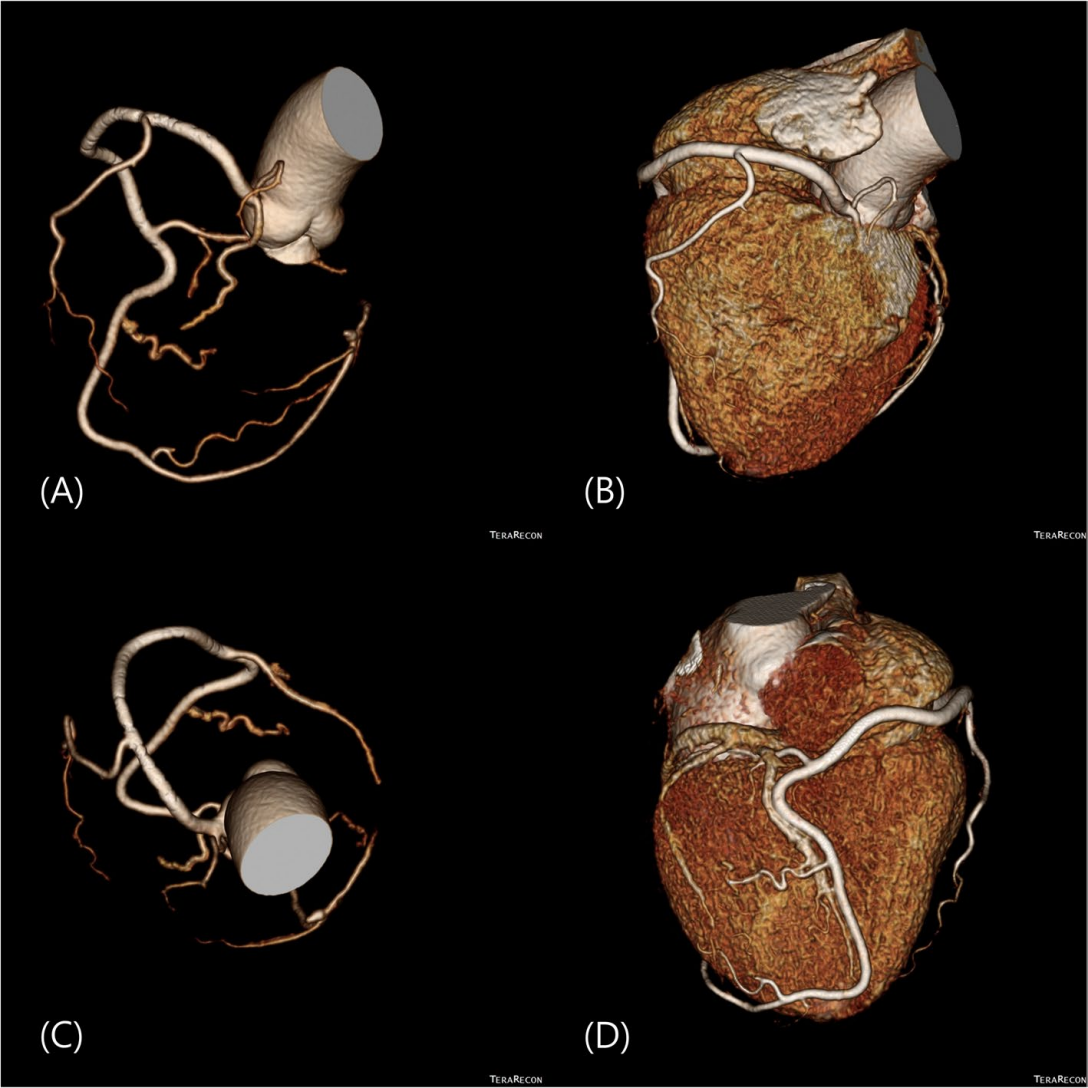
Coronary computed tomography angiography of the patient. **A** Volume-rendered coronary tree. **B** Volume-rendered reconstruction. **C** Cranial view of coronary tree (**D**) Posterior view of the coronary artery. The posterior descending artery courses through the heart’s apex and goes straight as the left anterior descending artery

A 65-year-old woman without past medical history was referred to our hospital for evaluation of dyspnea on exertion. Her chest X-ray showed normal heart size, and her electrocardiogram showed normal sinus rhythm. Echocardiogram showed a mildly reduced ejection fraction (48%) with global hypokinesia of the left ventricle and mildly increased left atrial volume. Coronary computed tomography (CT) angiography showed a single coronary artery originating from the right sinus of Valsalva, without the left coronary artery (Fig. 1).

The single coronary artery is a rare congenital anomaly. A previous observational study shows the prevalence is approximately 0.024–0.066% [1–3]. Compare to the left type single coronary artery, the right type single coronary artery is extremely rare. To our best knowledge, this is the first case report in Korea.

In this patient’s CT results, no coronary atherosclerosis was found. However, because the single coronary artery can increase the possibility of angina pectoris, myocardial infarction, congestive heart failure, or sudden death, we are now closely monitoring this patient to prevent such events.
